# A Case of Valproic-Acid Induced Hyperammonemic Encephalopathy

**DOI:** 10.7759/cureus.20380

**Published:** 2021-12-13

**Authors:** Kinza Iqbal, Hardhik Kummamuru, Naresh Dasari, Thoyaja Koritala, Nitesh K Jain, Keerti Deepika, Ramesh Adhikari

**Affiliations:** 1 Internal Medicine, Dow University of Health Sciences, Karachi, PAK; 2 Internal Medicine, QuickMDCare, Texas, USA; 3 Internal Medicine, Roger Williams Medical Center, Providence, USA; 4 Internal Medicine, Mayo Clinic, Mankato, USA; 5 Critical Care Medicine, Mayo Clinic, Mankato, USA; 6 Health Equity and Inclusion, Nemours Alfred I. duPont Hospital for Children, Wilmington, USA; 7 Hospital Medicine, Franciscan Health, Lafayette, USA; 8 Geriatrics, Brown University, Providence, USA

**Keywords:** adverse drug reaction, valproic acid, encephalopathy, hyperammonemia, valproate

## Abstract

Valproic acid (VPA), an antiepileptic medication, is known to cause hyperammonemia, which may be asymptomatic or can present with encephalopathy. VPA-induced hyperammonemic encephalopathy (VHE) is a serious but reversible condition, which requires high clinical suspicion for diagnosis. It may occur acutely or after chronic use of VPA. We present the case of a 44-year-old male who was on long-term VPA therapy for a seizure disorder. He presented to the emergency department with the complaint of two episodes of seizures two days before admission. On arrival, the patient was confused and tearful and was unable to recollect the events leading to the seizure. The initial complete metabolic panel, liver function tests, urinalysis, and serum VPA levels were observed to be normal. However, there was a marked elevation in ammonia levels. VPA was suspected to be the inciting agent of hyperammonemic encephalopathy, and, therefore, it was discontinued. The patient was started on oral lactulose and prescribed a different anti-seizure medication (i.e., lamotrigine). His ammonia levels decreased gradually, and his condition improved. Thus, it was concluded that the patient had developed VHE. At the time of discharge, he was stable and had no confusion or seizures. This case report evaluates his presentation and discusses the possible pathogenesis of VHE.

## Introduction

Valproic acid (VPA) is an antiepileptic medication that is frequently used in the treatment of epilepsy, migraines, and psychiatric disorders, including bipolar disorder and schizophrenia [1]. Although usually well-tolerated, VPA has been associated with a wide range of side effects ranging from nausea and vomiting to major adverse drug reactions such as pancreatitis and hepatotoxicity [2,3]. VPA-induced hyperammonemia is an important adverse drug reaction that may be asymptomatic or can present with VPA-induced hyperammonemic encephalopathy (VHE). VHE is typically characterized by nausea, vomiting, cognitive slowing, lethargy, coma, and seizures [4]. A timely diagnosis of VHE is essential in order to reverse the clinical symptoms by discontinuing VPA administration and lowering serum ammonia levels by administering lactulose or levocarnitine [4]. In the present case report, we describe a case of VHE in a patient with long-term use of valproate for seizure disorder and no liver dysfunction. The clinical characteristics and pathologic mechanisms of VHE are explained.

## Case presentation

A 44-year-old Caucasian male with a history of seizure disorder, benign hypertension, and nicotine addiction presented to the emergency department (ED) with two episodes of seizures two days prior to admission. He was incarcerated at a local jail 10 days prior to the ED visit. On arrival, the patient was confused and tearful and was unable to recollect the events leading to the seizures. He was admitted to the hospital for breakthrough seizures.

The patient was diagnosed with a seizure disorder at the age of 17 and was on seizure medications: divalproex extended-release (ER) (1,500 mg in the morning and 1,000 mg at night) and topiramate 50 mg BID (twice daily). Other home medications were metoprolol succinate 50 mg daily for benign hypertension and prazosin 2 mg QHS (every night at bedtime) for night terrors. The patient denied recent seizures before the current episode. He had a 22 pack-year history of nicotine consumption and denied alcohol consumption or the use of recreational drugs. He denied missing any medications or taking excessive medications during the incarceration. He had no family history of seizures, and his parents had died of coronary artery disease.

The initial complete metabolic panel was normal, except for a slight elevation of chloride, i.e., 108 mmol/L (normal = 98-107 mmol/L) and alkaline phosphatase, i.e., 106 U/mL (normal = 34-104 U/ml). The initial lactic acid levels were noted to be normal, i.e., 1.2 mmol/L (normal = 0.5-2.0 mmol/L). However, the ammonia levels were elevated significantly, i.e., 174 umol/L (normal = 16-53 umol/L). Liver function tests and urinalysis were observed to be normal. Urine drug screen for drugs, including amphetamines, barbiturates, cocaine, phenyl cyclohexyl piperidine, benzodiazepines, opioids, and cannabinoids, was normal. Tylenol and salicylate levels were within normal limits. On the day of admission, computed tomography (CT) of the head (without contrast) was performed, which revealed intact intracranial contents and no bleeding or edema. Electrocardiogram (EKG) findings revealed a normal sinus rhythm, with a rate of 65 beats/minute, a normal axis, and a QTc interval of 414-430 ms. VPA levels, which were investigated a day after the admission, were found to be within normal therapeutic limits of 88.40 ug/mL (normal = 50.00-100.00 ug/mL). Carnitine levels were found to be within the normal range, i.e., 60 umol/L (normal = 34-86 umol/L). However, topiramate levels were noted to be low, i.e., 2.0 ug/mL (normal = 5.0-20.0 ug/mL). Electroencephalogram (EEG) findings showed no actual epileptiform changes, and findings were consistent with a degree of encephalopathy.

The initial differential diagnosis was breakthrough seizures as the patient's VPA levels were therapeutic and possibly lowering the seizure threshold with a subtherapeutic level of topiramate. Another differential diagnosis was possible drug-induced encephalopathy due to divalproex sodium (i.e., Depakote). According to the existing literature, VPA can disrupt the urea cycle, leading to hyperammonemia. Therefore, we had a high suspicion that VPA may be the inciting agent of the patient’s condition. A teleneurologist was consulted, and VPA was discontinued and the patient was started on oral lactulose.

The patient had hyperammonemia and signs of encephalopathy with no hepatic dysfunction. Discontinuation of VPA and administration of lactulose improved his mentation and gradually reduced his ammonia levels, thus confirming our diagnosis of VHE. He was discharged on a different antiepileptic medication, i.e., lamotrigine. At the time of discharge, he was stable and had no new seizures or confusion.

## Discussion

VPA-induced hyperammonemia is a reversible adverse drug reaction that may occur at therapeutic or supratherapeutic doses of VPA in patients with normal liver function [5]. Chopra et al. performed a literature review of 30 cases of VHE, of which 16 occurred within six weeks after starting VPA therapy [2]. However, the diagnosis of VHE occurring several years after the initiation of VPA therapy can be more challenging for clinicians, as VHE may be unexpected. Some cases of VHE, occurring many years after the initiation of VPA therapy, have been reported in the existing literature [6-9]. Our case report adds to the literature; our patient used 1,000 mg to 15,00 mg VPA for 27 years without any drug-induced complications; VHE occurred after 27 years, with no identifiable precipitant or recent acute medical illness. Therefore, clinicians should consider the possibility of VPA-induced encephalopathy in patients with unexplained altered mental status and normal liver function, regardless of the duration of VPA therapy [6].

Although the exact mechanism of VPA-induced hyperammonemia remains unclear, it is thought to disrupt the urea cycle, either directly or through its metabolites. The rate-limiting enzyme of the urea cycle, carbamoyl-phosphate synthetase I (CPS-I), requires N-acetyl glutamate (NAG) as its allosteric activator. An enzyme, N-acetyl glutamate synthetase (NAGS), forms N-acetyl glutamate (NAG) from acetyl CoA and glutamate [2]. Direct inhibition of CPS-I by VPA is thought to be the primary mechanism of VPA-induced hyperammonemia [2,10]. Furthermore, VPA metabolism occurs in the liver via glucuronic acid conjugation and mitochondrial beta- and cytosolic omega-oxidation. VPA requires carnitine for entry into mitochondria; chronic use, high doses, or overdose can cause carnitine deficiency leading to VPA-induced toxicity [1]. Moreover, the metabolites of VPA, such as propionate and 4-en-VPA, are involved in the pathogenesis of VPA-induced hyperammonemia. Propionate decreases hepatic NAG levels, which, in turn, inhibits CPS-I, leading to hyperammonemia. The other metabolite of VPA, en-VPA, decreases the acetyl CoA available for NAG production by forming valproyl-CoA (VP-CoA) (2). VP-CoA has also been implicated to directly inhibit the activity of NAGS, resulting in VPA-induced hyperammonemia [2,10]. Prior literature suggests that genetic defects in two essential urea cycle enzymes, CPS-I and ornithine transcarbamylase (OTC), could contribute to VPA-induced hyperammonemia [1,10]. This explains why VPA-induced hyperammonemia occurs only in a subset of patients [10].

Another plausible mechanism is drug-drug interactions. Drugs such as phenytoin, carbamazepine, and phenobarbital can affect the function of CPS-1 [1]. Topiramate, an anticonvulsant, is known to inhibit both the urea cycle and glutamine synthetase activity. Prior cases have identified a recent addition of topiramate to induce hyperammonemic encephalopathy in patients previously on VPA therapy [2]. Moreover, there have been cases of the addition of VPA inciting hyperammonemic encephalopathy in patients on topiramate therapy [11]. Topiramate is believed to synergistically enhance the mechanism of VPA and lead to hyperammonemia [11]. In our patient, there was no recent change in the anti-epileptic drug regimen. He was on long-term concurrent VPA (since the age of 17) and topiramate therapy (most of the years, but it was unclear when it was started). Our case is unique as the patient tolerated VPA as well as topiramate for many years without any side effects and developed VHE without any recent precipitant. Moreover, coadministration of risperidone and VPA raises the levels of free VPA as risperidone competes with VPA for binding to albumin, which can subsequently disrupt the urea cycle and lead to VHE [12]. Duman et al. conducted a prospective, cross-sectional study to evaluate the risk factors for VPA-induced hyperammonemia. In the 11 epileptic patients included in the study, the concurrent use of multiple antiepileptic drugs was suggested to induce the CYP450 system. This may have increased the required daily VPA dose to obtain a target VPA level in the blood, consequently increasing the risk of hyperammonemia [13].

Hyperammonemia causes central nervous system (CNS) toxicity. In hyperammonemia, elevated levels of glutamine in the astrocytes are due to the excess conjugation of glutamate with ammonium to form glutamine and the inhibition of the release of glutamine from the astrocytes exposed to ammonium. High levels of glutamine increase the intracellular osmolarity of the astrocytes, drawing water into the astrocytes and resulting in astrocyte swelling and cerebral edema [1,2]. The high glutamate levels have also been linked with the activation of the N-methyl-D-aspartate receptor (NMDA) type of glutamate receptors, leading to the pathogenesis of hyperammonemic encephalopathy [2].

VPA-induced hyperammonemia ranges from subclinical cases to potentially fatal VHE. Although no treatment changes are needed in patients with asymptomatic hyperammonemia, it is important to closely monitor them for symptoms of VHE. There are multiple treatment modalities for patients diagnosed with VHE, the primary treatment being the discontinuation of VPA. It usually results in full recovery in most psychiatric patients [2]. According to a recent literature review, VHE was resolved in 10 of the 17 patients simply by the withdrawal of VPA [1]. Lactulose, a nonabsorbable disaccharide, is used as first-line therapy for hepatic encephalopathy. It lowers the production and absorption of ammonia; therefore, it is commonly used to correct VPA-induced hyperammonemia [2,14]. Carnitine supplementation is often used to correct the raised plasma ammonia levels by binding to VPA, thus de-inhibiting the urea cycle [2]. Other measures include hemodialysis, vitamin B, intravenous hydration, and mannitol [1].

Our patient developed VHE at therapeutic levels of VPA. Cessation of VPA and lactulose administration resolved his symptoms and gradually decreased his ammonia levels. VHE was confirmed by the absence of hepatic dysfunction and the symptom resolution on VPA discontinuation. He was discharged on a different antiepileptic medication, i.e., lamotrigine.

## Conclusions

Although hyperammonemia in the setting of hepatic failure is common in hospitals, acute or chronic VPA therapy is a relatively less common cause of hyperammonemia. VPA-induced hyperammonemia may be asymptomatic or can present with VHE. VHE is typically characterized by nausea, vomiting, cognitive slowing, lethargy, coma, and seizures. In the present case, VHE occurred in a patient with long-term VPA use for a seizure disorder, with normal hepatic function. Immediate discontinuation of VPA and administration of lactulose resulted in symptom resolution and a gradual decline in ammonia levels, confirming our diagnosis of VHE. The exact pathogenesis of VHE remains unclear; however, disruption of the urea cycle is thought to be the central mechanism behind VHE. Clinicians should consider the possibility of VHE in patients with unexplained altered mental status and normal hepatic function, regardless of the duration of VPA therapy. A timely diagnosis is essential to prompt effective treatment, thus ensuring the patient's safety and decreasing the length of hospitalization and the cost of care in hospitals.
